# KMUP-1 Attenuates Endothelin-1-Induced Cardiomyocyte Hypertrophy through Activation of Heme Oxygenase-1 and Suppression of the Akt/GSK-3β, Calcineurin/NFATc4 and RhoA/ROCK Pathways

**DOI:** 10.3390/molecules200610435

**Published:** 2015-06-05

**Authors:** Shu-Fen Liou, Jong-Hau Hsu, You-Ting Chen, Ing-Jun Chen, Jwu-Lai Yeh

**Affiliations:** 1 Department of Pharmacy, Chia-Nan University of Pharmacy and Science, Tainan 717, Taiwan; 2 Department of Paediatrics, Kaohsiung Medical University Hospital, Kaohsiung 807, Taiwan; 3 Department of Paediatrics, Faculty of Medicine, College of Medicine, Kaohsiung Medical University, Kaohsiung 807, Taiwan; 4 Department and Graduate Institute of Pharmacology, School of Medicine, College of Medicine, Kaohsiung Medical University, Kaohsiung 807, Taiwan; 5 Department of Marine Biotechnology and Resources, National Sun Yat-sen University, Kaohsiung 804, Taiwan

**Keywords:** KMUP-1, endothelin-1, cardiac hypertrophy, reactive oxygen species, heme oxygenase-1

## Abstract

The signaling cascades of the mitogen activated protein kinase (MAPK) family, calcineurin/NFATc4, and PI3K/Akt/GSK3, are believed to participate in endothelin-1 (ET-1)-induced cardiac hypertrophy. The aim of this study was to investigate whether KMUP-1, a synthetic xanthine-based derivative, prevents cardiomyocyte hypertrophy induced by ET-1 and to elucidate the underlying mechanisms. We found that in H9c2 cardiomyocytes, stimulation with ET-1 (100 nM) for 4 days induced cell hypertrophy and enhanced expressions of hypertrophic markers, including atrial natriuretic peptide and brain natriuretic peptide, which were all inhibited by KMUP-1 in a dose-dependent manner. In addition, KMUP-1 prevented ET-1-induced intracellular reactive oxygen species generation determined by the DCFH-DA assay in cardiomyocytes. KMUP-1 also attenuated phosphorylation of ERK1/2 and Akt/GSK-3β, and activation of calcineurin/NFATc4 and RhoA/ROCK pathways induced by ET-1. Furthermore, we found that the expression of heme oxygenase-1 **(**HO-1), a stress-response enzyme implicated in cardio-protection, was up-regulated by KMUP-1. Finally, KMUP-1 attenuated ET-1-stimulated activator protein-1 DNA binding activity. In conclusion, KMUP-1 attenuates cardiomyocyte hypertrophy induced by ET-1 through inhibiting ERK1/2, calcineurin/NFATc4 and RhoA/ROCK pathways, with associated cardioprotective effects via HO-1 activation. Therefore, KMUP-1 may have a role in pharmacological therapy of cardiac hypertrophy.

## 1. Introduction

Cardiovascular disease is a severe problem that remains a leading cause of death in the United States [1]. Pathological cardiac hypertrophy, a maladaptive process during cardiac dysfunction or hypertension, may increase the morbidity and mortality of cardiovascular diseases. At the cellular level, the hypertrophic process of cardiomyocytes consists of multiple events, including gene transcription and protein translation/synthesis, which are regulated by multiple signaling cascades. 

Mechanisms underlying cardiac hypertrophy involve a wide array of molecular pathways. Emerging evidence suggests that Ca^2+^ handling systems, such as calcineurin, Ca^2+^/calmodulin-dependent protein kinases (CaMK), and nuclear factor of activated T-cells (NFAT), have complex but important roles in pathophysiology of hypertrophy [2]. For example, it has been recently shown that activated CaMKII and CaMKIV have different roles in the modulation of hypertrophic responses. In cardiomyocytes, CaMKII can induce the hypertrophic-responsive gene atrial natriuretic peptide (ANP) [3]. Intriguingly, Santulli *et al.* found that CaMKIV gene deletion can induce hypertension and cardiac hypertrophy through down-regulation of endothelial nitric oxide synthase (eNOS) activity [4]. Thus, novel pharmacologic strategy targeting calcium handling proteins may shed some light on the management of cardiac hypertrophy.

In addition to the calcium system, the signaling cascades of the mitogen-activated protein kinase (MAPK) family including extracellular signal-regulated kinase (ERK) and c-Jun *N*-terminal kinase (JNK) are involved in cardiac hypertrophy [5,6,7]. Recent studies also indicate that the RhoA/ROCK cascade plays a pivotal role in many aspects of cardiovascular function, including cardiac hypertrophy [8,9]. Therefore, pharmacological interventions of these signaling pathways may provide promising approaches in mitigating cardiac hypertrophy and heart failure, however data are currently lacking.

7-[2-[4-(2-Chlorophenyl)piperazinyl]ethyl]-1,3-dimethylxanthine (KMUP-1), a novel xanthine-based derivative developed by our laboratory, has been demonstrated to inhibit activity of phosphodiesterase (PDE)3, 4, 5, and degradation of cAMP and cGMP, resulting in vasodilatation [10]. We have previously shown that KMUP-1 possesses pleotropic effects including calcium modulation and anti-proliferation in various cells, partly through regulations of the MAPK pathway [11,12,13,14]. More importantly, our recent study has demonstrated it can mitigate pulmonary artery hypertension with attenuation of right ventricle hypertrophy by suppressing endothelin-1 (ET-1), inactivating RhoA/ROCK, and enhancing eNOS expression [15]. However, whether these cardioprotective effects of KMUP-1 on pulmonary arterial hypertension can be extrapolated in the context of ET-1-induced cardiac hypertrophy is unknown. Therefore, the purpose of this study was to investigate whether KMUP-1 has an anti-hypertrophic effect on ET-1-induced cardiomyocyte hypertrophy, and to elucidate the potential mechanisms.

## 2. Results and Discussion

### 2.1. KMUP-1 Inhibited ET-1-Induced Cardiomyocyte Hypertrophy

**Figure 1:**
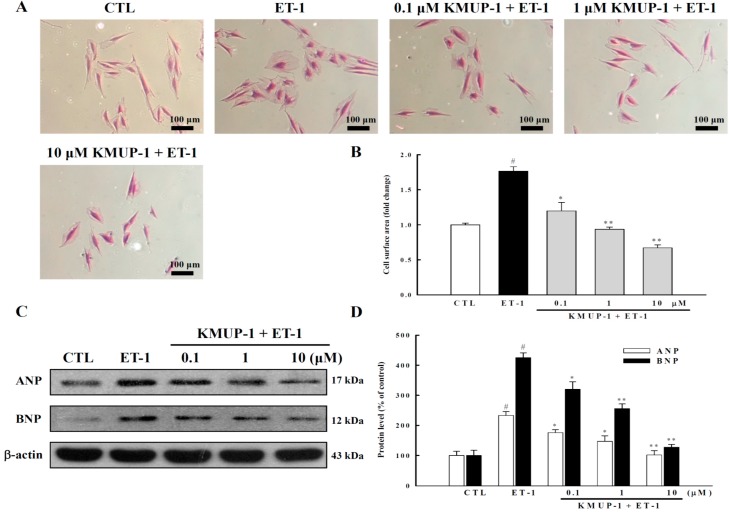
Effects of different doses of KMUP-1 on cell surface area, ANP and BNP protein expressions in myocytes treated with ET-1 (100 nM) for 4 days. (**A**) Representative micrographs of cardiomyocytes (200×); (**B**) Cell surface area was determined by planimetry, and >100 cells were analyzed per condition in six repeated experiments; (**C**,**D**) Expressions of ANP and BNP proteins. Each value represents the mean ± SEM of three independent experiments, with triplicate determinations in each experiment. # *p* < 0.01 compared with control; * *p* < 0.05, ** *p* < 0.01 compared with ET-1 alone.

Cardiac hypertrophy, marked by cardiomyocyte enlargement without proliferation, is an adaptive process in a variety of cardiovascular diseases [16]. In line with our recent study showing that KMUP-1 had anti-hypertrophic effects in the right ventricle in the model of pulmonary arterial hypertension *in vivo* [15], the present study further demonstrates that KMUP-1 can directly protect against ET-1-induced cardiomycyte hypertrophy *in vitro*. Our results showed that ET-1 significantly increased the surface area of cardiomyocytes by 77% (*p* < 0.05) (Figure 1A,B), and that KMUP-1 pretreatment markedly prevented the increase in cell surface area stimulated by ET-1 in a dose-dependent manner. 

Furthermore, we found that ET-1-induced hypertrophic responses were also characterized by upregulated expressions of fetal genes including atrial natriuretic peptide (ANP) and brain natriuretic peptide (BNP). These enhanced expressions were also inhibited by KMUP-1 dose-dependently (Figure 1C,D). Taken together, these results indicate that KMUP-1 inhibits ET-1-induced cardiomyocyte hypertrophy in both phenotypic and genotypic manners.

### 2.2. KMUP-1 Attenuated ET-1-Induced ROS Generation

**Figure 2:**
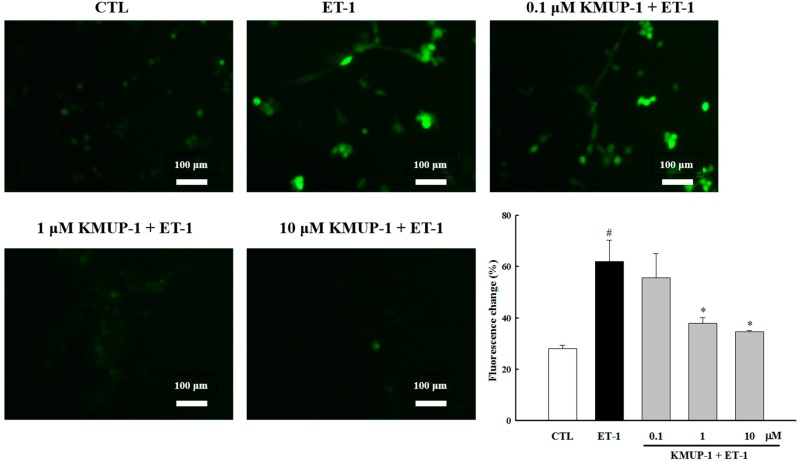
KMUP-1 inhibited ET-1-induced ROS production. Microscopic observation of DCF formation following ROS production (original magnification 200×). H9c2 cells were treated with different doses of KMUP-1 for 1 h, followed by co-treatment with 100 nM ET-1 for 3 h. Each value represents the mean ± SEM of three independent experiments, with triplicate determinations in each experiment. # *p* < 0.01 compared with control; * *p* < 0.05 compared with ET-1 alone.

It is well established that both reactive oxygen species (ROS) and reactive nitrogen species (RNS) play critical roles in modulation of myocardia function and development of heart diseases [17,18]. For example, various vasoactive peptides such as ET-1, angiotensin II, and norepinephrine have been shown to cause cardiomyocyte hypertrophy via ROS generation [19]. Indeed, several lines of evidence suggest that the inhibition of ROS generation by antioxidants can prevent cardiomyocyte enlargement induced by ET-1 [19,20,21]. Therefore, we further examined whether KMUP-1 could prevent ET-1-induced ROS production. As shown in Figure 2, ET-1 induced significant ROS production in H9c2 cells, and pretreatment with KMUP-1 attenuated ET-1-increased ROS production. These results suggest that the anti-hypertrophic effects of KMUP-1 may be partly attributed to suppression of ROS generation. 

### 2.3. Effects of KMUP-1 on MAPKs and Akt/GSK-3β Signaling

**Figure 3:**
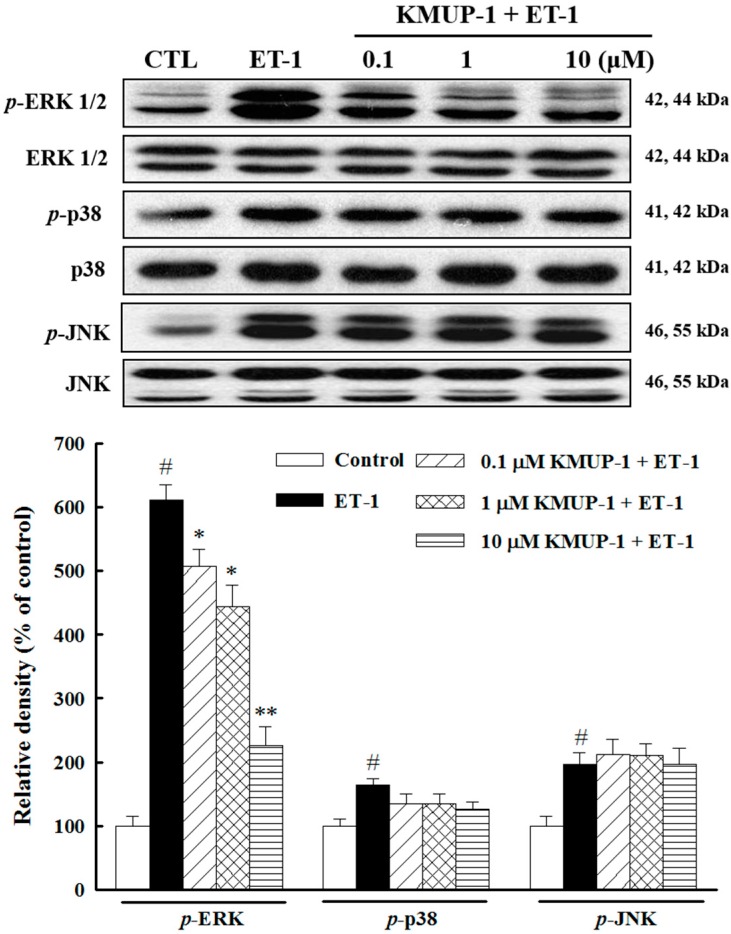
Effects of KMUP-1 on the activations of MAPKs pathway. H9c2 cells were treated with different doses of KMUP-1 for 1 h, followed by co-treatment with 100 nM ET-1 for 10 min. The protein expression levels of ERK1/2, p38, and JNK1/2 were significantly phosphorylated in ET-1-treated cardiomyocytes. KMUP-1 attenuated ET-1-induced phosphorylation of ERK 1/2, but not p38 and JNK1/2. The total MAPK levels were used as internal controls. Similar results are obtained from three independent experiments. # *p* < 0.01 compared with control; * *p* < 0.05, ** *p* < 0.01 compared with ET-1 alone.

Growth factors signaling through G protein-coupled receptors (GPCRs) (e.g., endothelin-1, norepinephrine, or angiotensin II) have emerged as potent mediators of cardiac myocyte hypertrophy. In fact, all three branches of the mitogen-activated protein kinase (MAPK) pathway are activated in response to GPCR stimulation in cardiac myocytes [22]. Therefore, we next investigated whether KMUP-1 modulates MAPKs pathway in ET-1-treated cardiomyocytes. As shown in Figure 3, after 10 min, ET-1 rapidly induced phosphorylation of ERK1/2, p38, and JNK by 6.1-fold, 1.6-fold, and 2.0-fold, respectively. Importantly, KMUP-1 markedly blocked only ERK1/2 activation in response to ET-1, without affecting the activation of p38 and JNK1/2. These data suggest that the inhibitory effects of KMUP-1 on cardiomyocyte hypertrophy are mediated mainly via the ERK pathway, which is consistent with our previous study showing that KMUP-1 inhibited isoprenaline-induced cardiac hypertrophy through inhibition of ERK1/2 phosphorylation [14].

**Figure 4:**
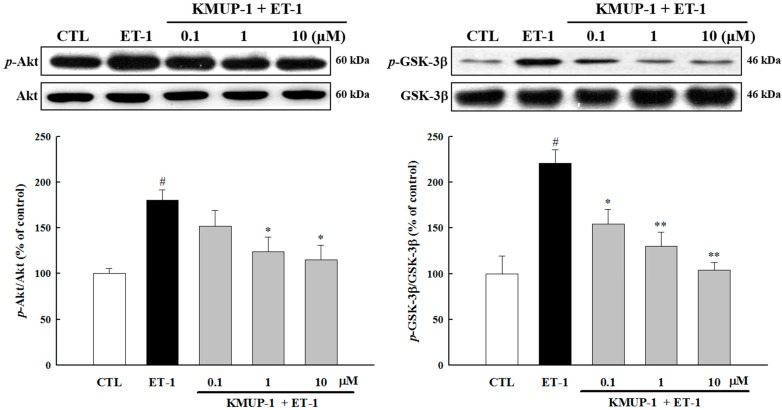
Effects of KMUP-1 on the Akt/GSK-3β pathway. H9c2 cells were treated with different doses of KMUP-1 for 1 h, followed by co-treatment with 100 nM ET-1 for 6 h. KMUP-1 prevented the phosphorylation of Akt and GSK-3β as seen in western blot analysis in ET-1-treated cardiomyocytes. Each value represents the mean ± SEM of three independent experiments, with triplicate determinations in each experiment. # *p* < 0.01 compared with control; * *p* < 0.05, ** *p* < 0.01 compared with ET-1 alone.

A growing body of evidence suggests that the Akt/GSK-3β pathway plays an important role in the development of cardiac hypertrophy and progression to heart failure [23,24]. To investigate its role in molecular mechanisms by which KMUP-1 mediates the hypertrophic effect induced by ET-1, we examined the activation state of Akt and its downstream mediator GSK-3β. As shown in Figure 4, up-regulation of *p*-Akt, and *p*-GSK-3β were observed after ET-1 treatment, and such effects were inhibited by pretreatment with KMUP-1. Taken together, our results suggest that both ERK1/2 and Akt/GSK-3β signaling pathways participate in the mechanisms underlying the anti-hypertrophic effects of KMUP-1.

### 2.4. KMUP-1 Suppressed ET-1-Activated Calcineurin/NFATc4 Signaling Pathway

**Figure 5:**
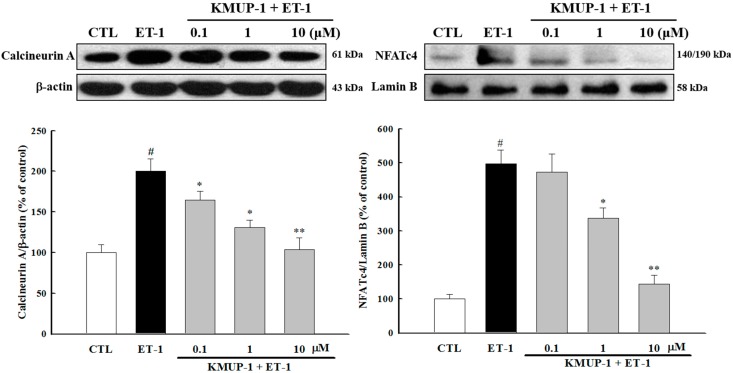
Effects of KMUP-1 on calcineurin-NFATc4 signaling. H9c2 cells were pretreated with different doses of KMUP-1 for 1 h, followed by cotreatment with 100 nM ET-1 for 6 h. KMUP-1 inhibited ET-1-induced calcineurin A expression and nuclear translocation of NFATc4. Each value represents the mean ± SEM of three independent experiments, with triplicate determinations in each experiment. # *p* < 0.01 compared with control; * *p* < 0.05, ** *p* < 0.01 compared with ET-1 alone.

Recently, the calcineurin/NFATc4 pathway has gained growing attention in the field of cardiovascular research. Indeed, the Ca^2+^/calmodulin (CaM)-activated calcineurin-NFAT signaling pathway has been shown to mediate the development of pathological cardiac hypertrophy [25]. As shown in Figure 5, we found that ET-1 significantly increased the expression of calcineurin A protein. Concomitantly, NFATc4 was found to have been transferred into the nucleus as evidenced by an increase in nuclear NFATc4 protein level by 5-fold (*p <* 0.01) after ET-1 stimulation. Furthermore, KMUP-1 significantly attenuated the ET-1-induced upregulation of calcineurin protein expression and nuclear translocation of NFATc4 (Figure 5). This *in vitro* evidence is consistent with our previous *in vivo* study in which KMUP-1 was found to inhibit the activation of the calcineurin-NFATc4 signaling pathway [14]. Emerging evidence suggests that calstabin2, a component of the cardiac ryanodine receptor (RyR2), has an important role in stabilizing RyR2 Ca^2+^ release channels and decreasing calcineurin activity [26,27]. Although we found that KMUP-1 significantly decreased the ET-1-induced expression of calcineurin, it is worthwhile to investigate in the future if calstabin2 has a role in the effects of KMUP-1 on the calcineurin/NFATc4 pathway.

### 2.5. Effect of KMUP-1 on ET-1-Induced RhoA and ROCK Activation

**Figure 6:**
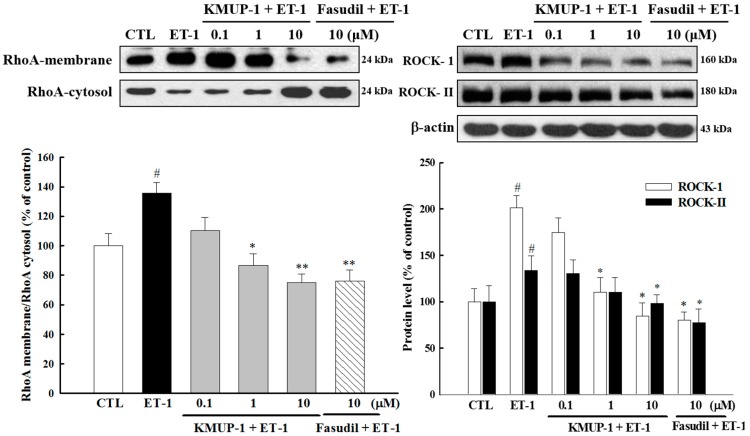
Effects of KMUP-1 on the RhoA/ROCK pathway. H9c2 cells were pretreated with different doses of KMUP-1 for 1 h, followed by co-treatment with 100 nM ET-1 for 30 min. The expression levels of Rho-kinase (ROCK-1 and ROCK-2) and ET-1-induced RhoA translocation were markedly attenuated by KMUP-1 and Fasudil (10 μM). Each value represents the mean ± SEM of three independent experiments, with triplicate determinations in each experiment. # *p* < 0.01 compared with control; * *p* < 0.05, ** *p* < 0.01 compared with ET-1 alone.

It has recently been demonstrated that the RhoA/ROCK signaling cascade may be a common pathway to mediate the development of cardiac hypertrophy and heart failure [28]. We therefore determined whether RhoA/ROCK signaling molecules participate in anti-hypertrophic effects of KMUP-1. We found that KMUP-1 and the ROCK inhibitor fasudil (10 μM) not only inhibited the ET-1-induced the translocation of RhoA from cell cytosol to the cell membrane, but also attenuated the expressions of ROCK-1 and ROCK-II isoforms (Figure 6). These results strengthen the notion that KMUP-1 prevents cardiomyocyte hypertrophy through ROCK inhibition similar as fasudil.

### 2.6. Effect of KMUP-1 on HO-1 Protein Expression

**Figure 7:**
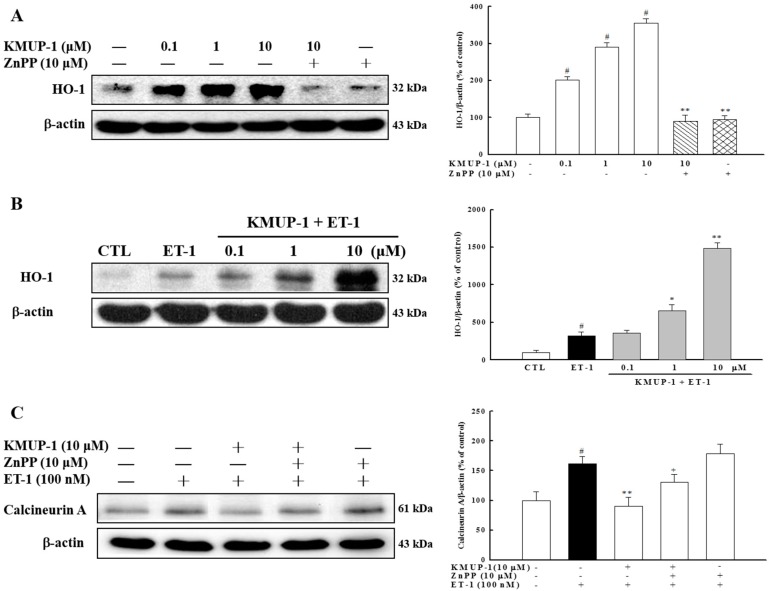
KMUP-1 treatment elevated the expression of HO-1 protein. (**A**) H9c2 cells were preincubated with ZnPP at 10 μM for 1 h, followed by co-treatment with KMUP-1 or (**B**) combination with 100 nM of ET-1. Western blot analysis was conducted to measure the induction of HO-1 protein; (**C**) The expression of calcineurin A was analyzed. # *p* < 0.01 compared with control; * *p* < 0.05, ** *p* < 0.01 compared with ET-1 alone. + *p* < 0.05 compared with KMUP-1 10 μM plus ET-1.

Another important physiological pathway that has recently been studied by many investigators for cardioprotection is heme oxygenase (HO). HO-1 is induced as a protective mechanism in response to various stimuli, and thus targeting this enzyme may be considered an emerging therapeutic strategy for protection against cardiac hypertrophy. For example, it has been reported that HO-1 inhibits cardiac hypertrophy through the MAPK signaling and calcineurin/NFAT pathways in ET-1 stimulated cardiomyocytes [22]. We therefore examined the role of HO-1 in the anti-hypertrophic effects of KMUP-1. As illustrated in Figure 7A, KMUP-1 induced the activation of HO-1, and the HO-1 inhibitor Zn-protoporphyrin (Znpp) abolished this effect. We also found that ET-1 induced the expression of HO-1 in cardiomyocytes, and that the addition of KMUP-1 led to higher levels of HO-1 protein in a dose-dependent manner (Figure 7B). To further understand whether the inhibitory effects of KMUP-1 on calcineurin expression were mediated by HO-1, we conducted experiments using the HO-1 inhibitor (Znpp). As shown in Figure 7C, Znpp attenuated the effects of KMUP-1 on calcineurin expression. These findings demonstrate for the first time that the inhibitory effect of KMUP-1 on calcineurin expression is mediated, at least in part, by the HO-1 pathway.

### 2.7. KMUP-1 Attenuated ET-1-Induced DNA Binding Activity of AP-1 

**Figure 8:**
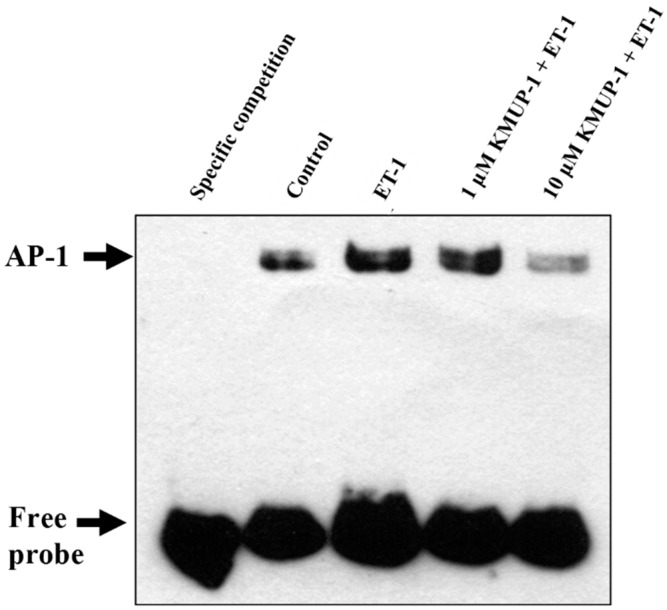
Effects of KMUP-1 on ET-1-induced AP-1 DNA binding activity. H9c2 cells were pretreated with different doses of KMUP-1 for 1 h, followed by co-treatment with 100 nM ET-1 for 6 h. The nuclear extracts were prepared to determine the binding activity of AP-1 by electrophoretic mobility shift assay. Similar results are obtained from three independent experiments.

Activator protein-1 (AP-1) contains c-fos and c-jun, which together form a heterodimer complex that plays a significant role in cardiac hypertrophy. A recent study showed that direct inhibition of AP-1 activity significantly decreased cardiac hypertrophy in myocardial tissue [29]. In addition, AP-1 transcription factors have been reported to be downstream nuclear mediators of multiple signaling pathways including PKC, the small G protein Ras, and MAPKs [30]. Recently, it has been shown that ET-1 induces c-fos gene expression via the generation of ROS in cardiomyocytes [20]. In order to investigate whether KMUP-1 inhibits the DNA binding activity of AP-1, we performed an EMSA assay using biotin-labeled double strand probes corresponding to AP-1 and its flanking sequence. As shown in Figure 8, ET-1 markedly induced the formation of the DNA-AP-1 complex, and this effect was significantly attenuated by KMUP-1. These findings suggest that deactivation of AP-1 may be one of the mechanism that contribute to the anti-hypertensive effects of KMUP-1. Apart from AP-1, NF-κB, is a ubiquitously expressed transcription factor involved in the pathogenesis of cardiac hypertrophy. Recent evidence has suggested a critical role of G protein-coupled receptor kinase 5 (GRK5) in mechanisms underlying NF-κB-mediated cardiac hypertrophy. Indeed, Sorriento *et al.* recently demonstrated that the RGS homology domain within the amino terminus of GRK5 (GRK5-NT) is able to inhibit NF-κB transcription activity, thus attenuating cardiac hypertrophy [31]. The present study did not address the issues of GRK5/NF-κB cascades, therefore future studies may be needed to investigate if their roles in anti-hypertrophic effects of KMUP-1.

## 3. Experimental Section

### 3.1. Materials

KMUP-1 hydrochloride (KMUP-1 HCl) was synthesized in our laboratory. ET-1, monoclonal antibodies against β-actin and HRP-conjugated secondary antibodies were purchased from Sigma-Aldrich (St. Louis, MO, USA). Zinc protoporphyrin (ZnPP) was purchased from Tocris Bioscience (Ellisville, MO, USA). Monoclonal antibodies against phospho-ERK, Akt, phospho-Akt and phospho-GSK3β (Ser^9^) were purchased from Cell Signaling Technology (Beverly, MA, USA). Polyclonal antibodies against ERK and phospho-JNK were purchased from Upstate Biotechnology (Lake Placid, NY, USA). Monoclonal antibody against JNK was purchased from R & D System (Minneapolis, MN, USA). Monoclonal antibody against phospho-p38 was purchased from Abcam System (Massachusetts, USA). Anti-HO-1, anti-GSK3β (Ser^9^), anti-calcineurin and anti-ROCK-II were purchased from BD Biosciences (San Diego, CA, USA). Anti-NFATc4, anti-p38 and anti-Rho A were purchased from Santa Cruz Biotechnology (Santa Cruz, CA, USA). Polyclonal antibody against ROCK-1 was purchased from Chemicon (Temecula, CA, USA). KMUP-1 HCl was dissolved in distilled water for experiments.

### 3.2. Cell Culture and Hypertrophy Induction

Cardiac H9c2 cells are a clonal heart muscle cell line, derived from embryonic rat hearts that retains many cardiomyocyte phenotypes [32]. All the cells used were derived from the same initial cell culture obtained from the American Type Culture Collection (ATCC, Rockville, MD, USA) and cultured in Dulbecco’s Modified Eagle Medium (DMEM) (Gibco BRL Life Technologies, Grand Island, NY, USA) supplemented with 10% fetal bovine serum (FBS) (Kibbutz Haemek, Israel) containing 100 U/mL penicillin G, 100 μg/mL streptomycin and 0.25 mg/mL amphotericin B, in a humidified atmosphere containing 5% CO_2_ at 37 °C. Before inducing hypertrophy, the medium was replaced with serum-free DMEM (0.1% FBS) for 24 h to achieve serum starvation and to synchronize cells into quiescence. Therefore, during hypertrophy induction by ET-1, low concentration (0.1%) FBS was used to minimize the effect of serum on cell hypertrophy. In the present study, serum starvation before each experiment was achieved by 0.1% FBS for 24 h to synchronize cells into quiescence.

### 3.3. Measurement of Cell Surface Area

The cells were seeded in 6-well plates at a density of 3 × 10^4^ cells per well. After 24 h culture, the medium was replaced with serum-free DMEM (0.1% FBS) for 24 h, followed by pretreatment with KMUP-1 for 60 min and then stimulation with ET-1 (100 nM) for 4 day to induce hypertrophy. After washing with phosphate-buffered saline, adherent cells were fixed with 1% glutaraldehyde for 30 min and stained with 0.1% crystal violet for 10 min at room temperature. The cell size was observed under an optic microscope (Olympus, Tokyo, Japan). Cell surface area was measured using Image-pro PLUS software. At least 20 randomly selected cells were measured for each N. The data shown represent the image analysis from three independent experiments.

### 3.4. Determination of Intracellular Reactive Oxygen Species

Production of ROS was determined using a DCFH-DA assay. H9c2 cells were incubated with ET-1 in the presence or absence of KMUP-1 for 3 h. In order to measure ROS production induced by ET-1, cells were stained with 10 μM of DCFH-DA for 30 min at 37 °C, and immunofluorescence was visualized using a digital camera attached to an inverted microscope. Photographic images were taken of five random fields. Immunofluorescence intensity was analyzed using Zeiss-Axio vision software. The data shown represent the image analysis from three independent experiments.

### 3.5. Western Blot Analysis

The cells were treated with indicated doses of KMUP-1 for the indicated times. The reactions were terminated by washing twice with cold PBS. The cells were then harvested and Western blot analysis was performed as previously described [14].

### 3.6. Electrophoretic Mobility Shift Assay (EMSA)

The protein content of the nuclear extracts of the H9c2 cells was determined using a Bio-Rad Protein Assay kit according to the manufacturer’s instructions. AP-1 consensus: 5′-CGCTTGATGAGTCAGC CGGAA-3′. The double-stranded oligonucleotides were end-labeled with 3′-biotin. EMSA was performed using a LightShift^®^ Chemiluminescent EMSA kit (Pierce Biotechnology), and the procedure was performed according to manufacturer’s instructions. Nuclear proteins (10 μg) were incubated with 1X binding buffer, 50 ng/μL poly (dI-dC), 2.50% glycerol, 0.05% NP-40, and 5 mM MgCl_2_ on ice for 20 min. 20 fmol biotin-labeled DNA was then added and mixed for another 20 min. The reaction mixture was analyzed using 8% nondenaturing polyacrylamide gel. The protein-DNA-biotin complexes were blotted onto Nylon membrane followed by UV cross-linking. The complexes were revealed with blocking buffer, horseradish peroxidase-conjugated streptavidin, subtract equilibrium buffer, and then exposed to the X-ray film.

### 3.7. Statistical Analysis

The results were expressed as mean ± SEM from at least three independent experiments. Student’s *t*-test was used to determine the significance of differences between two groups, and one-way ANOVA was used for multiple comparisons. A *p* value of less than 0.05 was considered statistically significant.

## 4. Conclusions

**Figure 9:**
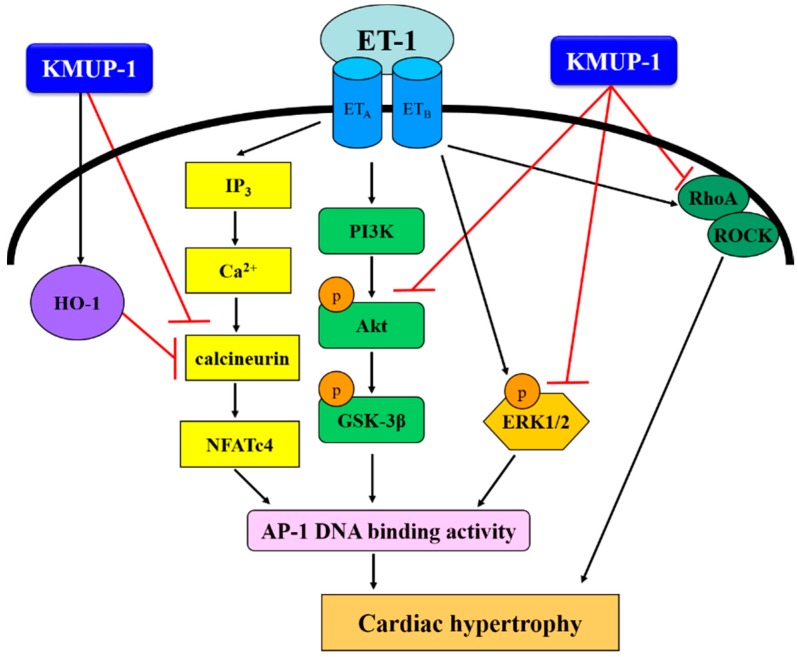
Proposed signaling pathways involved in the protective effects of KMUP-1 on ET-1-induced cardiomyocyte hypertrophy. The underlying mechanisms are associated with inhibition of p-ERK1/2, p-Akt, p-GSK-3β, calcineurin A, nuclear NFATc4 expression and Rho A translocation, subsequent AP-1 expression and up-regulation of HO-1.

Our results indicate that KMUP-1 attenuates cardiomyocyte hypertrophy induced by ET-1 through inhibiting the ERK1/2, calcineurin/NFATc4 and RhoA/ROCK pathways, with associated cardio-protective effects via activation of HO-1. The proposed mechanisms underlying the protective effects of KMUP-1 are shown in Figure 9.

Therefore, this study indicates that KMUP-1 might have a role in pharmacological therapy of cardiac hypertrophy. Further animal studies are needed to substantiate these novel findings.
